# Central Ohio Dental Association

**Published:** 1865-11

**Authors:** A. W. Maxwell


### Proceedings of Societies.

### CENTRAL OHIO DENTAL ASSOCIATION.

The organization of this Society was accomplished at a meeting held in the city of Mansfield, on the 5th day of September last, and the following officers elected: President, James Armstrong, Bucyrus; Vice President, M. De Camp, Mansfield; Recording Secretary, A. W. Maxwell, Galion; Corresponding Secretary, H. J. Cressinger, Ashland, and Treasurer, W. F. Semple, Fredericktown.

It was resolved to hold two regular meetings each year—on the 2nd Tuesday in May and the 2nd Tuesday in November.

The meeting, though small in numbers, passed off very pleasantly, and was characterized by a spirit of earnestness, and a determination that much good shall result from the organization of this Association, in the advancement of the interests of the Profession in this part of the State.

The First Semi-Annual Meeting will be held in Wooster, on the 14th of November next.

By a vote of the Society, a notice of the organization, was ordered to be sent to the Dental Register, and the Dental Cosmos. A. W. MAXWELL, Sect.

The First Semi-Annual Meeting of the Central Ohio Dental Association, will be held in Wooster, Wayne county, in Arcadome Hall, on Tuesday, the 14th day of November, 1865, at 10 o'clock A. M.

The organization of this Society was effected at a meeting held in the city of Mansfield, on the 5th day of September last, when it was resolved to hold two regular meetings each year—the annual, or principal business meeting, on the 2nd Tuesday in May, and the semi-annual, on the 2nd Tuesday in November. This call is for the first semi-annual meeting, and it is hoped that every Dentist who desires to aid in promoting the interests of the profession in this State, will be present. Essays will be read, and discussions had upon various subjects of interest to every member of the profession. A two days' session will be held. Let us have a large meeting and a good time.

October 10, 1865. A. W. MAXWELL, Sect.

